# Supplementary material for: The phonon thermal Hall angle in black phosphorus
Source: Nat Commun. 2023 Feb 23;14:1027. doi: 10.1038/s41467-023-36750-3 (PMC9950068; doi:10.1038/s41467-023-36750-3)
Supplement: Supplementary file 1 — Supplementary Information [file 41467_2023_36750_MOESM1_ESM.pdf]

Supplementary information for "The phonon thermal Hall angle in black phosphorus"

SUPPLEMENTARY NOTES

Supplementary Note 1: Sample details

The details of all samples are listed in Supplementary Table 1.

sample	l_x (mm)	l_z (mm)	l_y (mm)	measurements
#1-1	1.3	0.7	0.03	κ_{xx}, κ_{zz}, κ_{xz}, κ_{zx}
#1-2	0.6	0.7	0.02	ρ_{zz}, ρ_{zx}
#1-3	0.8	0.7	0.02	ρ_{xx}
#2-1	0.85	2.5	0.035	κ_{zz}, κ_{zx}
#2-2	3.0	1.3	0.07	κ_{xx}, κ_{xz}

Supplementary Table 1: Details of black phosphorus samples used in this work.

Supplementary Note 2: Processing the raw data

Supplementary Figure 1a shows the longitudinal and transverse temperature difference of sample #2-1 at 102 K under a heat power of 15.7 mW. With the field sweeping from -12 T to +12 T, the longitudinal temperature difference ΔT_i exhibits apparently symmetrical behavior, a large even signal accompanied by a tiny odd background. But the transverse temperature difference ΔT_j is predominantly asymmetrical: A large odd signal is accompanied by a small even background. To separate the signal from the background, we performed symmetric and asymmetric processing for the longitudinal and transverse temperature difference, respectively. For symmetric processing, we used $(\Delta T_i(+B)+\Delta T_i(-B))/2l$, here l is the length between two longitudinal thermocouples. For asymmetric processing we used $(\Delta T_j(+B)-\Delta T_j(-B))/2w$, here w is the length between two transverse thermocouples. The processed results are shown in Supplementary Figure 1b. The even component in the transverse response may come from either a lateral misalignment together with the magneto-thermal conductivity of the sample or from the magneto-thermopower of thermocouples. Note that the odd-to-even ratio is less than 1 mK/1.95 K in longitudinal response and more than 4 mK/0.36 K (i.e.) 20 times larger in the transverse response. This confirms that the asymmetrizied transverse signal is indeed the transverse thermal Hall gradient, which should be odd in magnetic field.

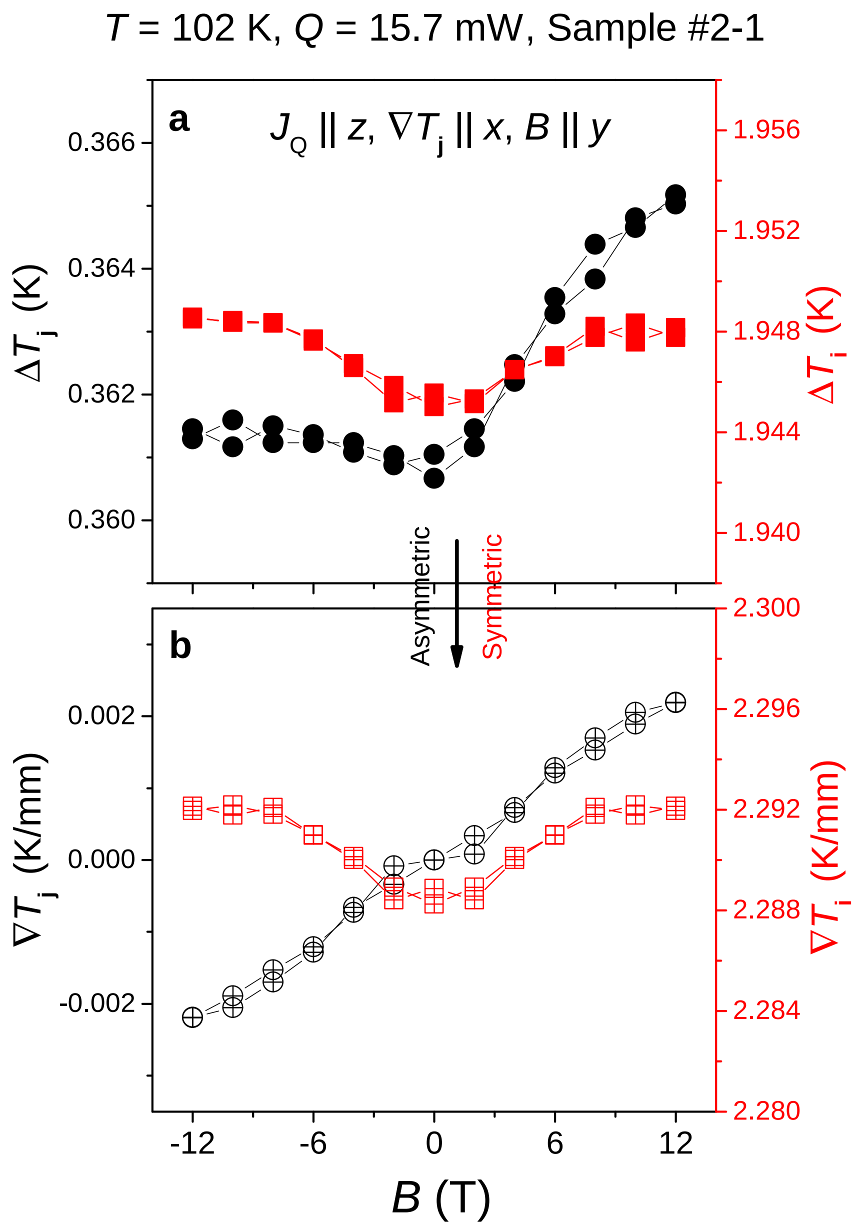

Supplementary Figure 1: Raw data and its processing. (**a**) The longitudinal and transverse temperature difference of sample #2-1 at 102 K under a heat power of 15.7 mW. (**b**) The results after the symmetric and asymmetric processing.

Supplementary Note 3: The anisotropic dielectric constant of black P

BP has an anisotropic dielectric constant. Nagahama *et al.* [1] experimentally determined its amplitude of along three different orientation by optical means and found that it is 13 along zigzag direction, 16.5 along armchair direction and 8.3 for out-of-plane direction.

Valagiannopoulos *et al.* [2] calculated the real and imaginary parts of the dielectric constant in black P. The

real components of their calculated values are in good agreement with what was measured [1] and show a modest anisotropy. Interestingly, there is a huge anisotropy in the imaginary component of the dielectric component along the two orientations (See the table). As a consequence, an electromagnetic wave is expected to be damped much less along the 'zigzag' direction compared to the 'armchair' direction.

	Re(armchair)	Re(zigzag)	Im(armchair)	Im(zigzag)
Theory [2]	18	14	0.45	0.04
experiment [1]	16.5	13	–	–

Supplementary Table 2: Measured and calculated components of the dielectric tensor in black P along different orientations.

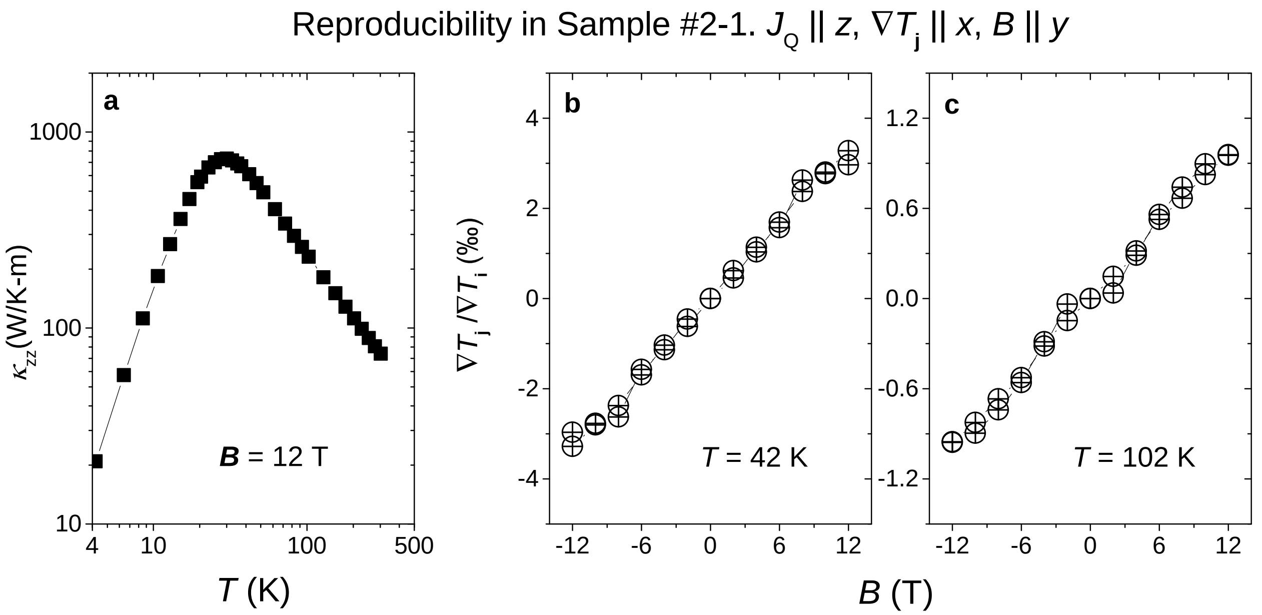

Supplementary Figure 2: Reproducibility of thermal Hall effect in sample #2-1. (**a**) The thermal conductivity of #2-1 along z-axis. (**b-c**) The thermal Hall angle of #2-1 at 42 K and 102 K.

Supplementary Note 4: Reproducibility

To ensure that the thermal Hall effect is intrinsic in black phosphorus, we repeated the measurements on other samples and used a different method. As seen in Supplementary Figure 2b and Supplementary Figure 3b, the thermal Hall effect was observed in two more samples #2-1 and #2-2. Their thermal Hall angle is anisotropic reflecting the anisotropy of the longitudinal thermal conductivity, as seen in Supplementary Figure 2a and Supplementary Figure

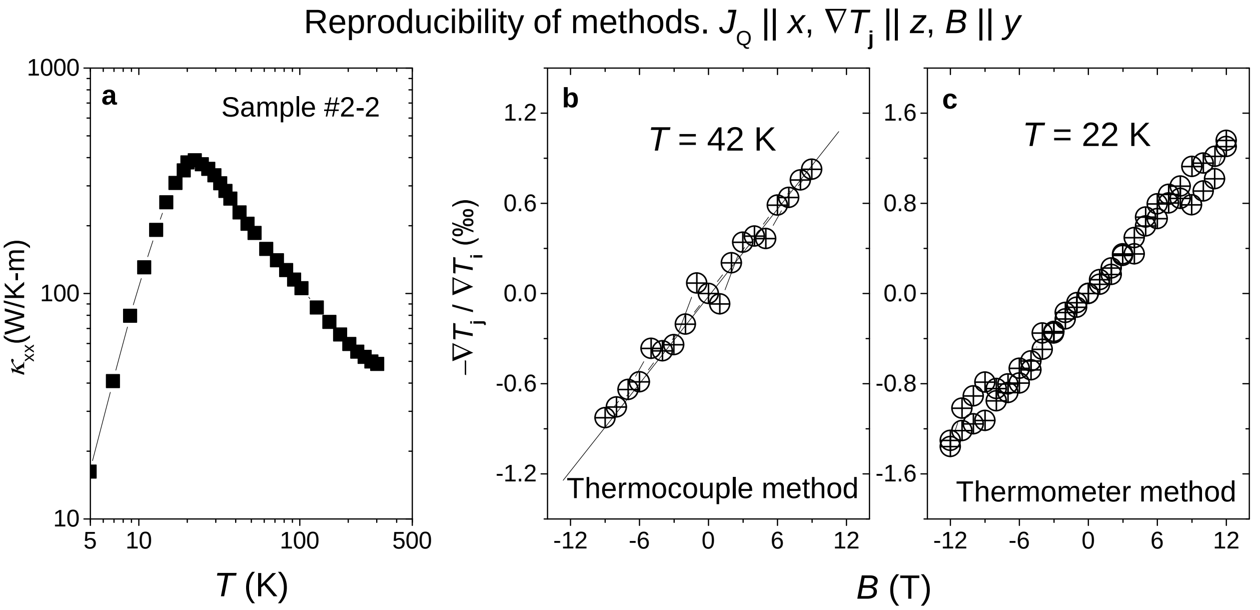

Supplementary Figure 3: Reproducibility of thermal Hall effect in different methods. (**a**) The thermal conductivity of #2-2 along x-axis by thermocouples method. (**b**) The thermal Hall angle of #2-2 at 42 K measured by the thermocouples method. (**c**) The thermal Hall angle of #2-2 at 22 K measured by the thermometers method.

3a. In addition, we measured sample #2-2 using resistive thermometers instead of thermocouples and found a similar result, as seen in Supplementary Figure 3c.

Supplementary Note 5: Role of electrons in thermal transport

Supplementary Figure 4a shows the resistivity of black phosphorus along different orientations. The electronic thermal conductivity κ_{xx}^e and κ_{zz}^e estimated from ρ_{xx} and ρ_{zz} through the Wiedemann-Franz law, is about 4 to 8 orders of magnitude smaller than the total thermal conductivity κ_{xx} and κ_{zz}, as seen in Supplementary Figure 4b, implying that phonons dominate the thermal transport in Black phosphorus.

Supplementary Figure 5a shows the temperature dependence of the three components of the electrical conductivity tensor. Supplementary Figure 5b compares the electrical and the thermal Hall angle. In the whole temperature range, the former is three orders of magnitude larger than the latter. This can generate a phonon drag thermal Hall effect [3] provided that there is large momentum exchange between electrons and phonons.

Supplementary Figure 5c compares the electronic thermal Hall conductivity estimated through the Wiedemann-Franz law and the measured thermal Hall conductivity. At low temperature they are separated by five orders of magnitude. On the other hand, at room temperature the difference is only one order of magnitude. Therefore, a

sizeable difference between the zero-electric-current and the zero-electric field thermal conductivities can arise due to the thermoelectric component of the thermal Hall conductivity. This feature was documented in detail in the case of metallic strontium titanate. It would explain the observed inequality between κ_{ij} and κ_{ji} near room temperature, where electrons matter most.

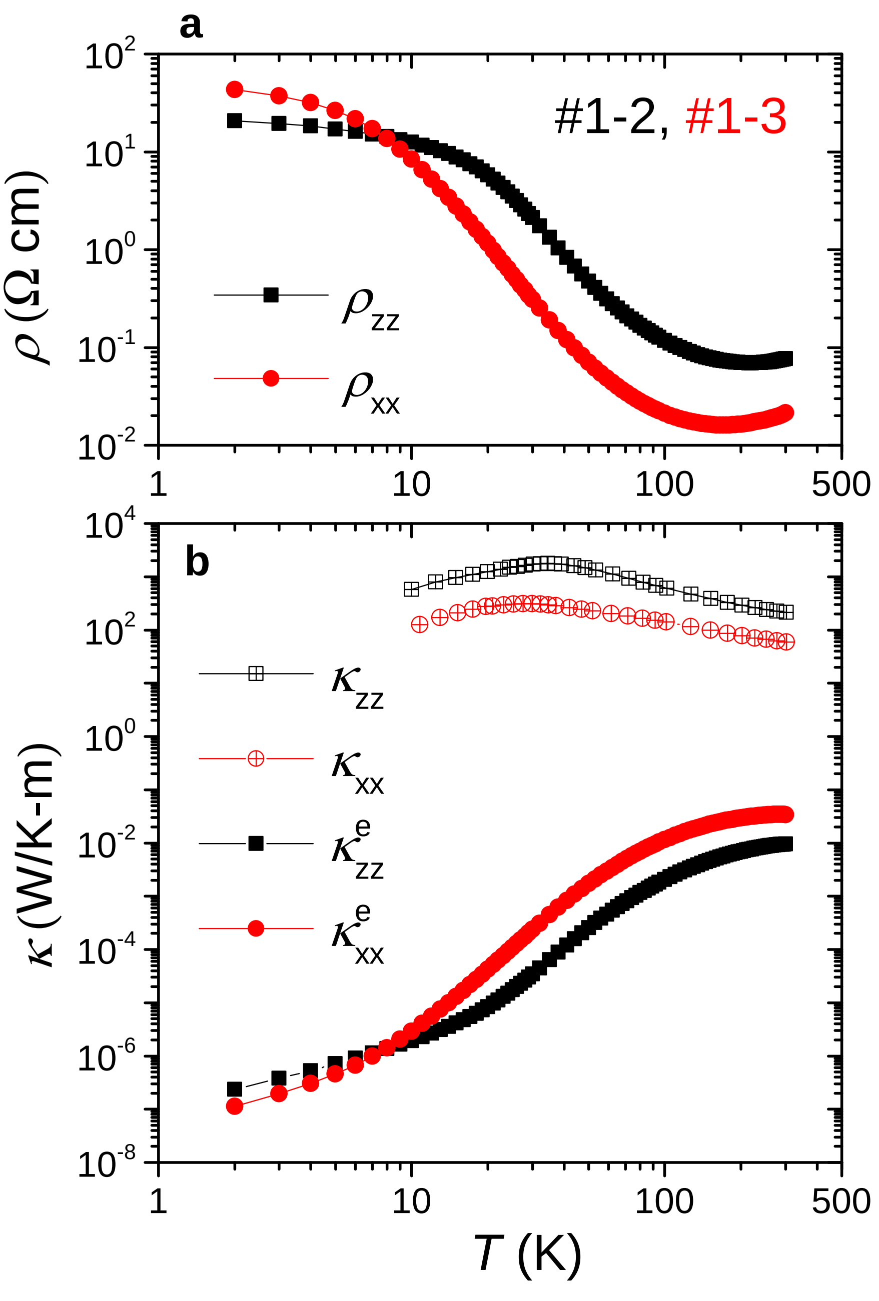

Supplementary Figure 4: Resistivity and electron thermal conductivity. (**a**) The ρ_{zz} measured in #1-2 and ρ_{xx} measured in #1-3. (**b**) The electron thermal conductivity κ^e_{xx} and κ^e_{zz} estimated through the Wiedemann-Franz law, compares with the total thermal conductivity κ_{xx} and κ_{zz} measured in #1-1.

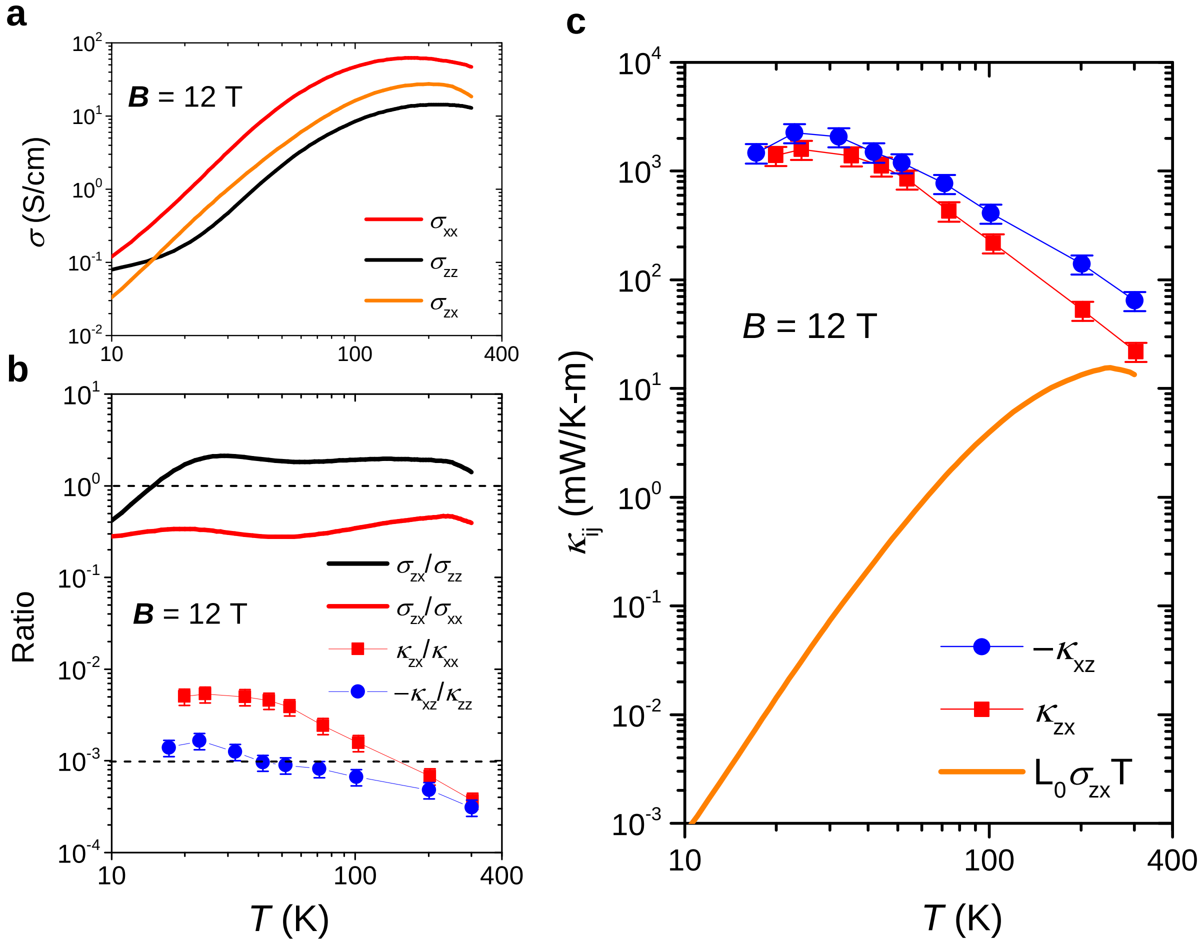

Supplementary Figure 5: Phonon drag and a thermoelectric component at high temperature. (**a**) Comparison of diagonal and off-diagonal electrical conductivity. (**b**) Comparison of the electrical Hall angle with their thermal counterparts. (**c**) Comparison of the electrical thermal Hall conductivity estimated through the Wiedemann-Franz law and the measured thermal Hall conductivity.

Supplementary Note 6: The mean free path of phonons and the sample thickness

Supplementary Figure 6 shows the mean free path of phonons in different BP crystals with different thicknesses [4]. It was extracted from the longitudinal thermal conductivity, the sound velocity and the specific heat. The mean-free-path shows a non-monotonic temperature dependence as a result of the Poiseuille flow of phonons. As the thickness of the sample increases, the absolute value of thermal conductivity enhances. This implies that at least a sub-set of phonons travel across the sample without collision.

Note that near the peak temperature ($\approx$ 30 K), the mean free path remains well below the sample thickness, which indicates that boundary scattering is not dominant. Interbranch phonon coupling is the most plausible source of

additional thermal resistivity.

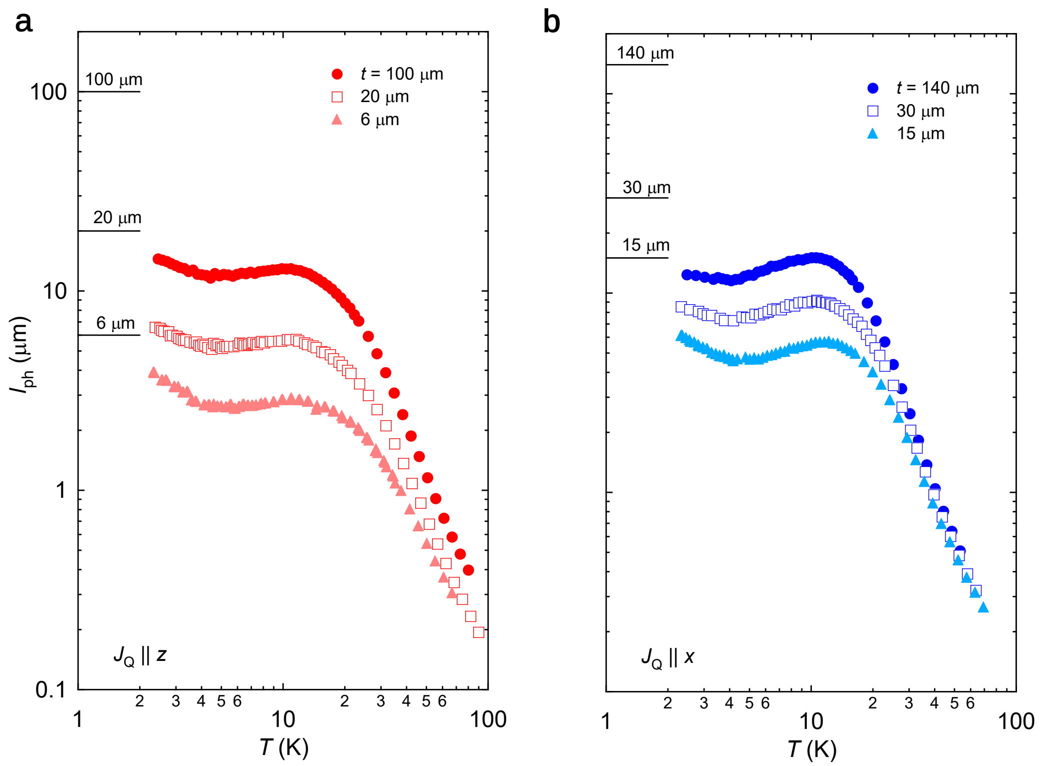

Supplementary Figure 6: Mean-free-path of phonons in BP. (**a-b**) The mean-free-path of phonons in BP for both orientations ($J_Q||z$ axis in **a** and $J_Q||x$ axis in **b**) for samples with different thicknesses [4]. The horizontal bars in the figure denote thickness of each sample. The mean-free-path increases with increasing thickness implying that a fraction of heat-carrying phonons are ballistic. But it remains well below the sample thickness indicating that there is another resistive process in addition to boundary scattering of acoustic phonons.

SUPPLEMENTARY REFERENCES

[1] Nagahama, T., Kobayashi, M., Akahama, Y., Endo, S. & Narita, S.-i. Optical determination of dielectric constant in black phosphorus. *Journal of the Physical Society of Japan* **54**, 2096–2099 (1985). URL `https://journals.jps.jp/doi/10.1143/JPSJ.54.2096`.

[2] Valagiannopoulos, C. A., Mattheakis, M., Shirodkar, S. N. & Kaxiras, E. Manipulating polarized light with a planar slab of

black phosphorus. *Journal of Physics Communications* **1**, 045003 (2017). URL https://dx.doi.org/10.1088/2399-6528/aa90c8.

[3] Jiang, S., Li, X., Fauqué, B. & Behnia, K. Phonon drag thermal Hall effect in metallic strontium titanate. *Proceedings of the National Academy of Sciences* **119**, e2201975119 (2022). URL https://www.pnas.org/doi/abs/10.1073/pnas.2201975119.

[4] Machida, Y. *et al.* Observation of poiseuille flow of phonons in black phosphorus. *Science Advances* **4**, eaat3374 (2018). URL https://www.science.org/doi/full/10.1126/sciadv.aat3374.
